# Nrf2、Keap1蛋白在104例肺腺癌中的表达及其临床病理特征

**DOI:** 10.3779/j.issn.1009-3419.2018.03.04

**Published:** 2018-03-20

**Authors:** 宇 肖, 翔 朱, 阳春 顾, 森 陈, 莉 梁, 宝山 曹

**Affiliations:** 1 100191 北京，北京大学第三医院肿瘤化疗与放射病科 Department of Medical Oncology and Radiation Sickness, Peking University Third Hospital, Beijing 100191, China; 2 100191 北京，北京大学第三医院病理科 Department of Pathology, Peking University Third Hospital, Beijing 100191, China

**Keywords:** Nrf2, Keap1, 肺腺癌, EGFR, EGFR-TKIs, Nrf2, Keap1, Lung adenocarcinoma, EGFR, EGFR-TKIs

## Abstract

**背景与目的:**

前期研究表明核因子E2相关因子2（nuclear factor erythroid-2-related factor 2, Nrf2）和Kelch样环氧氯丙烷相关蛋白1（Kelch-like ECH-associated protein 1, Keap1）的表达在肺癌患者中存在个体差异，其与化疗或表皮生长因子受体酪氨酸激酶抑制剂（epidermal growth factor receptor tyrosine kinase inhibitors, EGFR-TKIs）的疗效相关，但Nrf2及Keap1在不同驱动基因肺腺癌患者中的表达情况仍不清楚。本研究旨在探讨Nrf2、Keap1在肺腺癌患者中的表达与EGFR基因突变状态的关系及其对EGFR-TKIs疗效的影响。

**方法:**

应用免疫组化方法检测104例*EGFR*结果明确的肺腺癌患者，确定Nrf2、Keap1的表达情况，并分析其临床病理特征。

**结果:**

104例患者中Nrf2阳性率为71.2%，Keap1高表达率为34.6%；Nrf2阳性率与性别、分期和*EGFR*突变状态显著相关（*P* < 0.05），而与年龄、吸烟、分化程度、病理亚型无关（*P* > 0.05）；Keap1表达水平与年龄、性别、吸烟、病理亚型、肿瘤分化、EGFR突变状态等均无关（*P* > 0.05）；EGFR-TKIs治疗的患者无进展生存期（progression free survival, PFS）和总生存期（overall survival, OS）与Nrf2表达水平显著相关（*P* > 0.05），但与Keap1表达水平无关（*P* < 0.05）。Nrf2高表达组的中位PFS、OS显著低于低表达/阴性组（*P* < 0.05）。多因素分析表明Nrf2表达水平是EGFR-TKIs PFS和OS的独立预测因素。结论Nrf2阳性率与EGFR基因突变状态显著相关，Nrf2在EGFR突变肺腺癌患者中的表达水平与EGFR TKIs疗效显著相关，因此，Nrf2是预测EGFR TKIs疗效的理想指标和潜在的干预靶点。

## Abstract (en)

**Background and objective:**

There are significantly interindividual variations of the expression level of nuclear factor erythroid-2-related factor 2 (Nrf2) and/or Kelch-like ECH-associated protein 1 (Keap1) in our previous studies. It has been proven that Nrf2 or Keap1 is related to resistance of chemotherapeutic drugs and/or epidermal growth factor receptor tyrosine kinase inhibitors (EGFR-TKIs). However, the expression of Nrf2 and Keap1 in lung adenocarcinoma patients with different "driver gene" is not clear. The aim of this study is to investigate the protein expression level of Nrf2 and Keap1 in lung adenocarcinoma and to elucidate the correlation between Nrf2 or Keap1 expression and the status of *EGFR* gene mutation and to determine the effects of Nrf2 and Keap1 on the patients.

**Methods:**

Immunohistochemical analysis of Nrf2 and Keap1 in tumor specimens was performed in a total of 104 lung adenocarcinoma patients with the status of EGFR gene mutations or EGFR wide-type.

**Results:**

The Nrf2 positive rate was 71.2% and Keap1 high expression rate was 34.6% in 104 patients. The Nrf2 positive rate significantly correlated with gender, stage and status of *EGFR* gene mutation (*P* < 0.05), but not with age, smoking, differentiation and subtype of lung adenocarcinoma (*P* > 0.05). The high expression of Keap1 was not significantly correlated with gender, age, smoking, differentiation, subtype of lung adenocarcinoma and status of *EGFR* gene mutation (*P* > 0.05). The progression -free survival (PFS) and overall survival (OS) of the patients treated by EGFR-TKIs were significantly correlated with the expression level of Nrf2, but not with Keap1. The PFS and OS of the patients with Nrf2 high expression were significantly shorter than the patients with low/negative expression (*P* < 0.05). Furthermore, Nrf2 high expression was the independent predictive factor for EGFR-TKIs induced PFS and OS (*P* < 0.05).

**Conclusions:**

The Nrf2 positive rate significantly correlated with the status of *EGFR* gene mutation in lung adenocarcinoma. The Nrf2 high expression significantly correlated with PFS and OS of EGFR-TKIs. Therefore, Nrf2 may be a biomarker for predicting response of EGFR-TKIs and a potential target for overcoming resistance of EGFR-TKIs.

肺癌目前是世界范围内发病率和死亡率最高的恶性肿瘤^[1]^，其中80%为非小细胞肺癌（non-small cell lung cancer, NSCLC），约75%的NSCLC就诊时已属于中晚期^[2, 3]^。肺腺癌是NSCLC中最常见的一种病理类型，发现时亦多处于中晚期，药物治疗仍是其主要的治疗手段。尽管近年来随着对表皮生长因子受体（epidermal growth factor receptor, *EGFR*）基因功能的深入认识，EGFR酪氨酸激酶抑制剂（EGFR tyrosine kinase inhibitors, EGFR-TKIs）给*EGFR*突变肺腺癌带来了生存和生活质量的显著改善^[4-7]^，但不论是化疗，还是EGFR-TKIs，耐药仍是临床上亟待解决的问题。因此，探索药物疗效预测相关的分子指标是目前研究热点之一，这将有助于提高药物疗效、降低药物毒性，改善患者生活质量、减轻患者经济和心理压力。

核因子E2相关因子2[nuclear factor-erythroid 2 (NF-E2) p45-related factor 2, Nrf2]/Kelch样环氧氯丙烷相关蛋白-1（Kelch-like ECH-associated protein 1, Keap1）是细胞应对氧化应激和亲电性应激损伤的重要防御通路^[8, 9]^。生理状况下，Keap1结合Nrf2，并与E3泛素化连接酶结合，通过泛素化介导Nrf2蛋白降解，维持细胞浆内Nrf2较低水平。一旦细胞处于氧化应激或亲电应激状态，Keap1成为敏感的传感器，通过对自身半胱氨酸残基的修饰，阻止Nrf2降解，并促进Nrf2释放。累积的Nrf2进入细胞核内，激活抗氧化反应元件（antioxidant response element, ARE），进而激活ARE驱动的Ⅱ相药物代谢酶和抗氧化基因以及部分Ⅲ相药物转运蛋白表达，从而保护细胞免受氧化应激或亲电应激损伤^[8, 9]^。Nrf2/Keap1信号的异常激活不但参与肿瘤的发生、发展和转移^[10-12]^，而且还影响化疗药物^[9, 12, 13]^和EGFR-TKIs的疗效^[14, 15]^，Krall等在含有驱动基因的肺腺癌细胞中发现Keap1功能缺失会导致多种靶向药物耐药^[16]^。我们前期研究表明Keap1高表达者铂类药的疗效会减低^[17]^，EGFR突变患者中Nrf2高表达者EGFR-TKIs的疗效也出现降低^[14]^。因此，Nrf2/Keap1通路已成为肺癌预防和治疗的潜在靶点^[18]^，但Nrf2、Keap1在不同驱动基因肺腺癌患者中的表达情况和对相应治疗的影响，尚缺乏此方面报道。

本研究回顾性分析了2010年6月-2012年12月在北京大学第三医院接受过EGFR基因突变检测的肺腺癌患者104例，通过免疫组化方法，确定Nrf2、Keap1在EGFR突变型和野生型肺癌患者中的表达情况及其临床病理特征，并分析Nrf2、Keap1表达水平对EGFR-TKIs疗效的影响。

## 1 对象与方法

### 1.1 研究对象

选取2010年6月-2012年12月在北京大学第三医院接受过EGFR基因突变检测[通过扩增阻滞突变系统（amplification refractory mutation system, ARMS）或直接测序法]的肺癌患者。入组标准：组织学病理确诊为肺腺癌；具有明确的EGFR基因检测结果；有完善的影像学检查[胸腹部计算机断层扫描（computed tomography, CT）、头颅磁共振成像（magnetic resonance imaging, MRI）]可供肿瘤分期评价；有足够的组织标本可供免疫组化检测；预计生存期超过3个月的患者。排除标准：组织学病理非肺腺癌者（如鳞癌、大细胞癌、小细胞癌等）；缺乏足够的组织标本供免疫组化检测；治疗前分期评价不足的。

### 1.2 临床资料收集

采集并记录患者确诊时的临床资料，包括年龄、性别、吸烟状态、肿瘤分期、病理亚型、分化程度、*EGFR*基因突变结果和EGFR-TKIs治疗期间的影像学资料。

### 1.3 肿瘤分期及病理亚型诊断标准

肿瘤分期依据国际肺癌研究协会颁布的第7版分期标准^[19]^，肺腺癌病理亚型诊断依据国际肺癌研究协会/美国胸科学会/欧洲呼吸学会2011年颁布的关于肺腺癌的国际多学科新分类标准^[20]^。

### 1.4 远期疗效评价标准

远期疗效包括无疾病进展生存期（progression free survival, PFS）和总生存期（overall survival, OS）。PFS定义为从初次治疗开始至疾病进展或任何原因导致死亡的时间，OS定义为从初次治疗开始至死亡或随访终点时间。

### 1.5 随访

接受EGFR-TKIs治疗的患者通过定期来院或电话随访，随访开始时间为2010年7月，末次随访时间为2017年12月，最短随访时间3个月，最长75个月。

### 1.6 免疫组化检测Nrf2、Keap1蛋白表达

#### 1.6.1 实验方法

手术切除的组织标本或活检组织标本经10%甲醛固定后，常规石蜡包埋，切片，4 μm厚度。免疫组化采用SP法[兔抗人Nrf2抗体（ab31163）购于Abacm®公司，兔抗人Keap1抗体购于Proteintech®公司，对应的免疫组化二抗SP检测试剂盒购于北京中杉金桥生物技术有限公司，Nrf2及Keap1抗体均按1:100稀释]，应用PBS代替一抗作为阴性对照，按照试剂说明书进行操作。

#### 1.6.2 结果判定

采用单盲法阅片（病理医师不清楚临床资料），Nrf2抗原阳性反应可位于细胞浆和细胞核中，Keap1抗原阳性反应位于细胞浆内。参照Solis等^[21]^及曹宝山等^[14, 17]^前期研究结果，将细胞核染色强度和细胞核阳性比例乘积 > 0定义为Nrf2阳性，反之为阴性；将评分 > 100%，定义为Nrf2高表达，反之为低/不表达。将细胞染色强度和阳性细胞比例乘积 < 150%定义为Keap1低/不表达，反之为高表达。

### 1.7 统计学方法

应用SPSS 19.0统计学软件分析。率的比较采用卡方检验或*Fisher*精确检验；相关性检验采用*Pearson*检验；非参数检验采用*Wilcoxon*秩和检验，应用*Kaplan-Meier*方法进行生存分析，*Log-rank*检验差异性；多因素分析采用*Cox*多因素分析模型，逐步后退法（backward, walds）。全部统计检验均为双侧概率检验，检验水准*α*=0.05，以*P* < 0.05为差异有统计学意义。

## 2 结果

### 2.1 患者临床特征

符合入组条件的肺腺癌患者共104例，其中男性57例，女性47例，中位年龄为66岁（21岁-84岁），≥70岁者44例， < 70岁者60例；Ⅲ期和Ⅳ期患者共74例（71.1%）；中低分化者84例（80.8%）。腺泡型、实体型和贴壁型是本研究中最常见的病理亚型，分别为49例（47.1%）、22例（21.2%）及19例（18.3%）。本组患者中EGFR突变率为67.3%（70/104），其中外显子19缺失突变（19del）45例，外显子21 L858R点突变21例，其他突变4例（分别为外显子18 G719A和G724S突变各1例，外显子20 L815P和Y801C突变各1例），见表 1。相关分析表明，EGFR突变与肿瘤分期（*r*=0.176, *P*=0.073）及吸烟（*r*=-0.191, *P*=0.051）有一定相关趋势，但与年龄（*r*=0.057, *P*=0.562）、性别（*r*=0.139, *P*=0.161）、病理亚型（*r*=0.080, *P*=0.418）和病理分化程度（*r*=-0.097, *P*=0.328）等无相关。

**1:** 104例肺腺癌患者临床病理特征 Clinicopathologic characteristics of 104 lung adenocarcinoma cases

Clinical features	*n*	%
Age (yr)		
≥70	44	42.3
< 70	60	57.7
Range	21-84	
Median age	66	
Gender		
Female	47	45.2
Male	57	54.8
Smoking history		
Current/Former	44	42.3
Never	60	57.7
Staging		
Stage Ⅰ	24	23.1
Stage Ⅱ	6	5.8
Stage Ⅲ	25	24.0
Stage Ⅳ	49	47.1
Differentiation		
Low	18	17.3
Moderate	57	54.8
High	27	26.0
Other	2	1.9
Subtype of histology		
Lepidic	19	18.3
Acinar	49	47.1
Solid	22	21.2
Papillary	5	4.8
Other	9	8.7
Status of *EGFR* gene		
EGFR wide-type	34	32.7
*EGFR* mutation	70	67.3
19del	45	43.3
21L858R	21	20.2
Others (G719A, G724S, L815P, Y801C)	4	3.8
EGFR: epidermal growth factor receptor.		

### 2.2 Nrf2表达水平及其与患者临床特征间的关系

Nrf2表达在细胞核和细胞浆中均可以表达，且存在个体差异，与曹宝山等前期研究^[14]^一致。本组Nrf2阳性率为71.2%（74/104），Nrf2阳性水平在性别、分期和*EGFR*突变状态等组内存在显著差异（*P* < 0.05）。*EGFR*突变组中Nrf2阳性率为80.4%，显著高于EGFR野生型组Nrf2阳性率的50%（*P*=0.001）；女性患者中Nrf2阳性率为80.9%，显著高于男性组的63.2%（*P*=0.047）；分期越晚Nrf2阳性率越高（*P*=0.044）。但Nrf2表达水平在年龄、吸烟、肿瘤分化程度、病理亚型等组内无显著差异（*P* > 0.05），见表 2。Nrf2阳性率在19del和21L858R突变患者中分别为80.0%（36/45）和90.5%（19/21），二者无显著差异（*P*=0.287）。

**2:** Nrf2阳性率同肺腺癌患者临床特征间的关系 Relationship between positive Nrf2 and clinicopathologic characteristics of lung adenocarcinoma patients

Clinical features	*n*	Nrf2 postive rate [*n* (%)]^*^	*χ*^2^	*P*
Age (yr)	104		1.391	0.238
≥70	44	34 (77.3)		
< 70	60	40 (66.7)		
Gender	104		3.929	0.047
Female	47	38 (80.9)		
Male	57	36 (63.2)		
Smoking history	104		1.022	0.312
Current/former	44	29 (65.9)		
Never	60	45 (75.0)		
Staging	104		7.879	0.044
Stage Ⅰ	24	17 (70.8)		
Stage Ⅱ	6	6 (100.0)		
Stage Ⅲ	25	13 (52.0)		
Stage Ⅳ	49	38 (77.6)		
Differentiation			1.430	0.713
Low	18	12 (66.7)		
Moderate	57	42 (73.7)		
High	27	18 (66.7)		
Other	2	2 (100.0)		
Subtype of histology	104		0.651	0.954
Lepidic	19	14 (73.7)		
Acinar	49	35 (71.4)		
Solid	22	15 (68.2)		
Papillary	5	3 (60.0)		
Other	9	7 (77.8)		
Status of *EGFR* gene	104		11.013	0.001
EGFR wide-type	34	17 (50.0)		
*EGF*R mutation	70	57 (81.4)		
^*^Nrf2 positive refers to score > 0, the score was obtained by multiplying the intensity and reactivity extension values (range 0-300%).				

### 2.3 Keap1表达水平及其与患者临床特征间的关系

Keap1主要表达在细胞浆中，与曹宝山等前期研究结果^[17]^一致。本组Keap1高表达率为34.6%（36/104）。Keap1表达水平与肿瘤分期有一定相关趋势，但在年龄、性别、吸烟、病理亚型、分化程度、EGFR突变状态等组内无显著差异（*P* > 0.05），见表 3。

**3:** Keap1阳性率同肺腺癌患者临床特征间的关系 Relationship between Keap1 expression and clinicopathologic characteristics of lung adenocarcinoma patients

Clinical features	*n*	Keap1 postive rate [*n* (%)]^**^	*χ*^2^	*P*
Age (yr)	104		3.959	0.047
≥70	44	20 (45.5)		
< 70	60	16 (26.7)		
Gender	104		0.883	0.347
Female	47	14 (29.8)		
Male	57	22 (38.6)		
Smoking history	104		0.264	0.608
Current/former	44	14 (31.8)		
Never	60	22 (36.7)		
Staging	104		6.981	0.072
Stage Ⅰ	24	5 (20.8)		
Stage Ⅱ	6	0 (0.0)		
Stage Ⅲ	25	10 (40.0)		
Stage Ⅳ	49	21 (42.9)		
Differentiation			3.848	0.312
Low	18	5 (27.8)		
Moderate	57	18 (31.6)		
High	27	13 (48.1)		
Other	2	0 (0.0)		
Subtype of histology	104		1.874	0.775
Lepidic	19	5(26.3)		
Acinar	49	16 (32.7)		
Solid	22	10 (45.5)		
Papillary	5	2 (40.0)		
Other	9	3 (33.3)		
Status of *EGFR* gene	104		0.604	0.437
EGFR wide-type	34	10 (29.4)		
*EGFR* mutation	70	26 (37.1)		
^**^: Keap1 positive refers to score≥150%, the score was obtained by multiplying the intensity and reactivity extension values (range 0-300%).				

### 2.4 Nrf2表达水平及其与EGFR-TKIs治疗患者临床特征间的关系

本研究中38例患者接受了EGFR-TKIs治疗，无*EGFR*基因突变者2例，资料不全者6例，30例*EGFR*突变接受EGFR-TKIs治疗且资料齐全。30例患者中，17例为吉非替尼，6例为厄洛替尼，7例为埃克替尼；Nrf2阳性率为86.7%（26/30），Nrf2高表达率为40.0%（12/30）。Nrf2的高表达率与性别有关，女性组为63.2%（12/19），显著高于男性组的0（0/11）（*P*=0.001），但在年龄、吸烟、肿瘤分化、肿瘤分期和*EGFR*突变类型等组内无显著差异（*P* > 0.05），见表 4。

**4:** Nrf2高表达率同接受EGFR-TKIs治疗肺腺癌患者临床特征间的关系 Relationship between Nrf2 nuclear high expression and clinicopathologic characteristics of lung adenocarcinoma patients treated by EGFR-TKIs

Clinical characterstic	*n*	Nrf2 nuclear high expression rate [*n* (%)]^***^	*χ*^2^	*P*
Gender	30		11.579	0.001
Male	11	0 (0.0%)		
Female	19	12 (63.2%)		
Age (yr)	30		1.094	0.296
< 70	14	7 (50.0%)		
≥70	16	5 (31.3%)		
Smoking history	30		2.500	0.114
Current/former	10	2 (20.0%)		
Never	20	10 (50.0%)		
Differentiation	30		0330	0.848
Low	4	2 (50.0%)		
Moderate	16	6 (37.5%)		
High	9	3 (33.3%)		
Staging	30		1.693	0.193
Ⅲb	9	2 (22.2%)		
Ⅳ	21	10 (47.6%)		
*EGFR* mutation	30		0.010	0.919
19 deletion	20	7 (35.0%)		
21 L858R	8	4 (50.0%)		
Others	2	1 (50.0%)		
^***^: Nrf2 high expression refers to score > 100%, the score was obtained by multiplying the intensity and reactivity extension values (range 0-300%).				

### 2.5 生存分析

30例EGFR-TKIs治疗的患者中，相关分析表明PFS与Nrf2高表达呈负相关（*r*=-0.527, *P*=0.003），而与年龄、性别、吸烟、肿瘤分化、肿瘤分期、Keap1表达水平等无关（*P* > 0.05）。Nrf2高表达组患者的PFS显著低于不/低表达组，分别为（4.6±2.9）个月和（20.2±16.1）个月（*P*=0.003）。Kaplan-Meier生存分析表明：①Nrf2阳性组和阴性组间的OS无显著差异，中位OS分别为24.0个月（95%CI: 15.3-32.7）和50.0个月（95%CI: 0.0-106.9）（*P*=0.861），图 1A。②Keap1高表达组和不/低表达组间的OS无显著差异，中位OS分别为23.0个月（95%CI: 16.6-29.4）和50.0个月（95%CI: 0.0-101.5）（*P*=0.801），图 1B。③Nrf2高表达组的OS显著低于不/低表达/组，中位OS分别为15.0个月（95%CI: 10.0-20.0）和60.0个月（95%CI: 44.8-75.2）（*P*=0.001），图 1C。④患者OS与*EGFR*突变类型无关。19del、21L858R和罕见突变组的中位OS分别50.0个月（95%CI: 3.5-96.5）、20.0个月（95%CI: 6.0-34.0）和3.0个月（*P*=0.695），图 1D。⑤患者的OS与分期无关，Ⅲb期和Ⅳ期两组的中位OS分别为60.0个月（95%CI: 4.3-115.7）和24.0个月（95%CI: 13.0-35.0）（*P*=0.604），图 1E。

**1:**
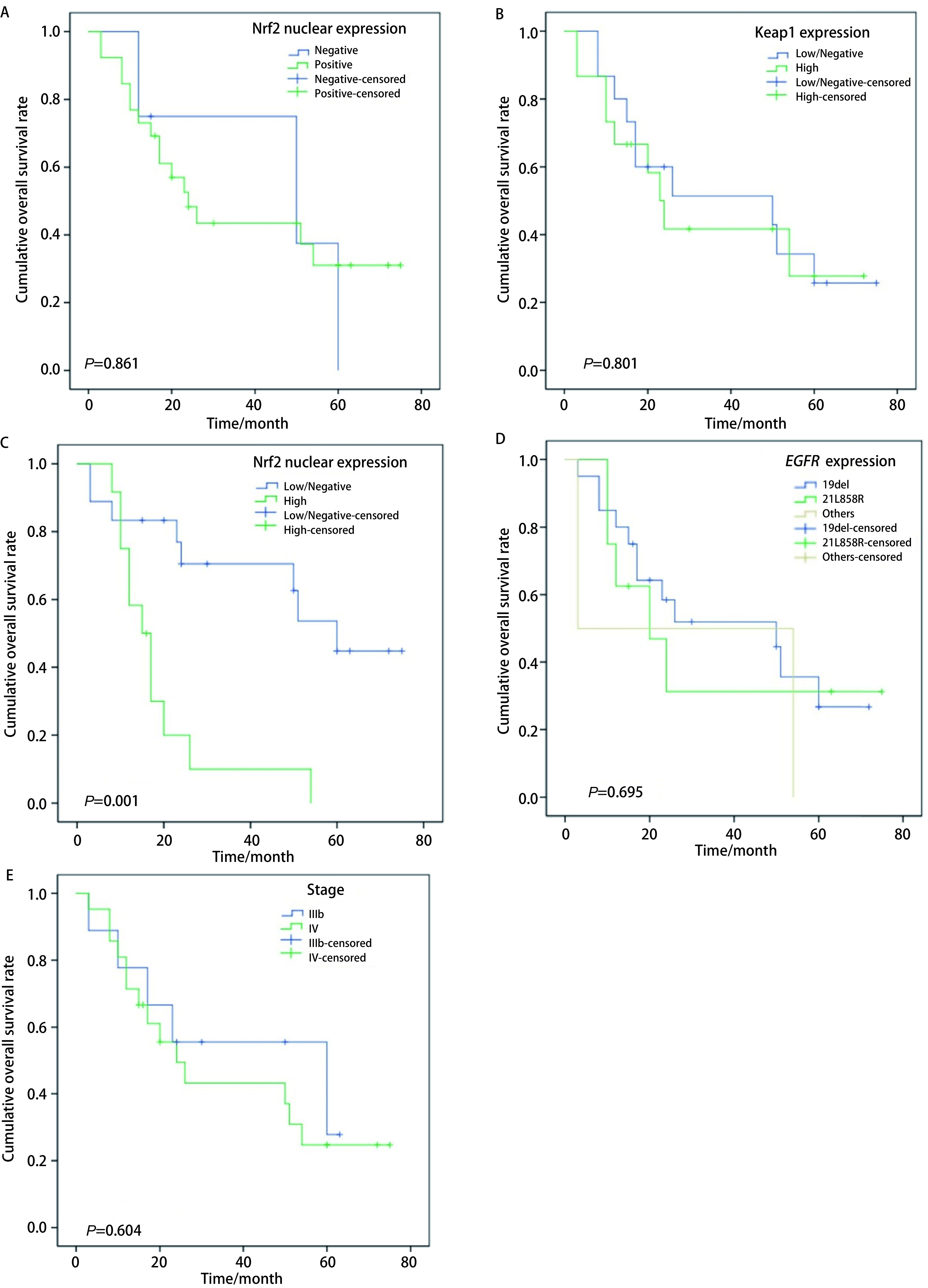
EGFR-TKIs治疗患者总生存期在不同组间的变化。A：Nrf2阳性组和阴性组患者的中位OS无显著差异；B：Keap1高表达组和不/低表达组患者的中位OS无显著差异；C：Nrf2高表达组中位OS显著低于不/低表达组；D：*EGFR*基因不同突变组的中位OS无显著差异；E：Ⅲb期和Ⅳ期患者的中位OS无显著差异。 Overall survival (OS) of patients treated by EGFR-TKIs in different groups. A: The median OS of lung adenocarcinoma patients between Nrf2 positive group and Nrf2 negative group were similar; B: The median OS of lung adenocarcinoma patients between Keap1 high expression group and Keap1 low/negative expression group were similar; C: The median OS of lung adenocarcinoma patients of Nrf2 high expression group was significantly lower than that of Nrf2 low/negative group; D: The median OS of lung adenocarcinoma patients among different *EGFR* mutation groups were not significantly different; E: The median OS of lung adenocarcinoma patients between stage Ⅲb and stage Ⅳ were similar.

### 2.6 *Cox*回归分析

在校对患者*EGFR*基因突变状态、Keap1表达水平、分期、性别和Nrf2高表达等因素后，多因素分析表明Nrf2高表达是EGFR-TKIs PFS（*P*=0.002）和OS（*P*=0.009）的独立预测因素。Keap1表达水平对PFS和OS无显著预测价值（*P* > 0.05），见表 5。

**5:** 多因素分析EGFR-TKIs特异性生存的预后因素(*n*=30) *Cox* regression analysis of the disease-specific survival with EGFR TKIs (*n*=30)

Variable	Regression coefficient *β*	Standard error	Wald	*P* value	Exp(B)	95%CI
Progression-free survival						
*EGFR* mutation	0.598	0.362	2.729	0.099	1.818	0.895-3.696
Keap1	0.034	0.432	0.006	0.937	1.035	0.443-2.414
Stage	0.092	0.474	0.036	0.846	1.096	0.433-2.778
Gender	-0.914	0.546	2.803	0.094	0.401	0.137-1.169
Nrf2 high expression	2.139	0.681	9.863	0.002	8.487	2.234-32.239
Overall survival						
*EGFR* mutation	-0.814	0.415	0.196	0.658	0.832	0.369-1.876
Keap1	0.202	0.535	0.142	0.706	1.224	0.428-3.494
Stage	-0.298	0.571	0.273	0.602	0.742	0.243-2.272
Gender	-0.525	0.764	0.472	0.492	0.592	0.133-2.643
Nrf2 high expression	2.016	0.771	6.836	0.009	7.505	1.656-34.007

## 3 讨论

肺腺癌目前是NSCLC中最常见的病理类型，已发现*EGFR*、*ALK*、*ROS1*等多种驱动基因^[22]^，其中EGFR在亚裔患者中是最常见的一种^[6, 7, 22]^。本研究中*EGFR*突变率67.3%（70/104)，19del和21L858R突变占整体突变的94.3%，*EGFR*突变倾向于发生在分期晚和不吸烟的患者中，与既往研究结果类似^[4, 6, 7]^；但本研究中*EGFR*突变的发生与性别和病理亚型无关，且19del突变显著多于21L858R突变，与前期研究结果不同^[23]^。产生差异的原因在于病例非连续入组，存在选择偏倚。EGFR-TKIs是进展期*EGFR*基因敏感突变患者一线治疗的首选药物，但耐药仍是临床面临的难题。Nrf2/Keap1通路是细胞应对氧化应激和亲电性应激损伤的重要防御通路，Nrf2、Keap1在肺癌中与药物耐药和肿瘤发生、发展和转移密切相关。曹宝山等前期研究表明Nrf2/Keap1通路与肺癌化疗和靶向治疗疗效有关，其中Keap1高表达会降低铂类药物疗效^[17]^，而Nrf2高表达会减低EGFR-TKIs的疗效^[14]^。近期Krall等发现Keap1功能缺失会导致EGFR-TKIs耐药^[16]^。但Nrf2和Keap1的表达水平与*EGFR*基因突变状态是否相关，且Keap1的表达水平是否会影响EGFR-TKIs的疗效，目前缺乏此方面的临床研究。

本研究通过免疫组化方法检测104例肺腺癌患者，结果发现Nrf2阳性率为71.2%，其中在*EGFR*基因突变组Nrf2阳性率为80.4%，显著高于*EGFR*基因野生型组的50.0%（*P*=0.001），但Nrf2阳性率在19del和21L858R两突变组间无显著差别（*P* > 0.05）。在*EGFR*基因突变组中Nrf2阳性率与前期小样本研究结果类似（77.4%）^[14]^，但远高于Solis等^[21]^报道的26.0%的阳性率。本研究还发现Nrf2阳性率与分期有关，分期越晚Nrf2阳性率越高（*P*=0.044），与朱翔等研究结果^[14]^类似。本研究结果与Solis等^[21]^产生差异的原因为：①Solis等^[21]^研究的患者以早期为主，晚期患者数量不足1/4；②Yamadori等^[15]^研究发现EGFR信号活化可上调Nrf2表达水平，因此在*EGFR*敏感基因突变患者中，EGFR通路活化是导致Nrf2阳性表达率增高原因之一；③Solis等^[21]^报道的23例*EGFR*基因突变患者中无Nrf2阳性表达，此种差异或许与肿瘤分期、种族等因素有关。本研究中Keap1高表达率为34.6%（36/104），高于前期研究中的26.0%（13/50）^[17]^，Keap1的表达水平与肿瘤分期有一定相关趋势，但与*EGFR*突变状态无关。与前期研究产生差异的原因或许在于前期研究中含有40%鳞癌患者及样本量较小^[17]^。上述结果可通过前瞻性研究得到进一步的验证。

本研究在含有*EGFR*基因突变并且接受EGFR-TKIs治疗的患者中发现：EGFR-TKIs的疗效与Nrf2表达水平显著相关，Nrf2高表达组的PFS和OS显著低于Nrf2不/低表达组，多因素分析表明Nrf2表达水平是EGFR-TKIs治疗患者PFS和OS的独立预后因素。这与朱翔等研究结果相一致^[14]^，且与Yamadori等^[15]^在含有*EGFR*基因敏感突变的细胞株中发现的现象一致，即Nrf2激活会导致EGFR-TKIs耐药。这或许是因为：Nrf2一旦激活，其进入细胞核中，进而激活ARE调控的药物解毒酶和代谢酶，从而促进细胞增殖、抑制细胞凋亡^[9, 24]^。但在本研究中，患者的PFS和OS与*EGFR*基因不同突变位点无关，这与Jackman等^[25]^研究结果不同，即外显子19del突变人群的OS优于21858R突变的人群。可能与本研究样本量小有关。

本研究Keap1高表达组与不/低表达组的两组患者在PFS和OS均无显著差异，提示Keap1表达水平或许与EGFR-TKIs疗效无关。但Krall等^[16]^发现Keap1功能消失会激活Nrf2，进而导致厄洛替尼的耐药^[16]^。两项研究结果之间产生差异的原因在于：①Keap1是细胞内环境敏感的传感器，Keap1表达情况随着外界环境刺激发生改变^[9]^，其真正发挥作用具有一定滞后性；②Krall等^[16]^研究利用的基因敲除技术，使得Keap1功能真正消失，而本研究中Keap1的表达水平并不能代表*Keap1*基因的功能。因此，Keap1对EGFR-TKIs疗效的影响进一步需要在基因水平进行验证。

本研究通过回顾性研究分析，发现Nrf2阳性率在*EGFR*基因突变的患者显著升高，Nrf2高表达者EGFR-TKIs的PFS和OS差，多因素分析表明Nrf2表达水平是EGFR-TKIs PFS和OS的独立预测因子，但Keap1表达水平与*EGFR*基因突变状态及EGFR-TKIs疗效无关。综上可见，Nrf2在含有*EGFR*突变患者中或许是预测EGFR-TKIs疗效理想的分子指标，还是提高EGFR-TKIs疗效潜在的干预靶点。需要进一步扩大样本量进行前瞻性研究，验证Nrf2和Keap1的临床价值，并通过基因检测明确其具体机制。
